# Studying Food Reward and Motivation in Humans

**DOI:** 10.3791/51281

**Published:** 2014-03-19

**Authors:** Hisham Ziauddeen, Naresh Subramaniam, Victoria C. Cambridge, Nenad Medic, Ismaa Sadaf Farooqi, Paul C. Fletcher

**Affiliations:** ^1^Department of Psychiatry, University of Cambridge; ^2^Metabolic Research Laboratories, Wellcome Trust-MRC Institute of Metabolic Science, University of Cambridge; ^3^Cambridgeshire & Peterborough NHS Foundation Trust, University of Cambridge; ^4^West Anglia Comprehensive Local Research Network, Addenbrooke's Hospital

**Keywords:** Behavior, Issue 85, Food reward, motivation, grip force, willingness to pay, subliminal motivation

## Abstract

A key challenge in studying reward processing in humans is to go beyond subjective self-report measures and quantify different aspects of reward such as hedonics, motivation, and goal value in more objective ways. This is particularly relevant for the understanding of overeating and obesity as well as their potential treatments. In this paper are described a set of measures of food-related motivation using handgrip force as a motivational measure. These methods can be used to examine changes in food related motivation with metabolic (satiety) and pharmacological manipulations and can be used to evaluate interventions targeted at overeating and obesity. However to understand food-related decision making in the complex food environment it is essential to be able to ascertain the reward goal values that guide the decisions and behavioral choices that people make. These values are hidden but it is possible to ascertain them more objectively using metrics such as the willingness to pay and a method for this is described. Both these sets of methods provide quantitative measures of motivation and goal value that can be compared within and between individuals.

**Figure:**
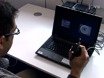


## Introduction

The study of food reward processing in humans has received a significant impetus from the rising concerns about the obesity epidemic. As the route to obesity in most obese individuals is through increased energy intake over and above metabolic need^1^, it is important to understand the drivers and mechanisms of overconsumption. Prevailing models consider this overconsumption to be a form of nonhomeostatic or ‘hedonic' eating, *i.e.* consumption that is driven not by homeostatic needs but by the rewarding aspects of the food(s) consumed^2^. However this is a complex phenomenon and the homeostatic and hedonic/reward systems are overlapping and interactive. Further there are several factors that affect nonhomeostatic eating that do not directly relate to hedonic aspects of the food such as portion size or the variety of foods available^3^. Nevertheless it is important to be able characterize and measure the different aspects of food reward.

Berridge and colleagues have described three components of reward: liking, wanting, and learning. These three components are linked but are dissociable in terms of their underlying neural systems. Liking refers to the hedonic impact of a reward and wanting is the motivation for the reward. Learning comprises the associations with and predictions about the reward. These components are further distinguished into two subcomponents, core or implicit, and conscious or explicit. Liking is comprised of ‘liking' which refers to core objective hedonic reactions, and the conscious subjective experience of pleasure. Similarly wanting consists of ‘wanting', the incentive salience of rewards and reward related cues, and the conscious subjective experience of desire for incentives as is normally understood by the term. The conscious, subjective experiences of liking and wanting are elaborated out of the core reactions by higher cognitive mechanisms. Lastly learning too consists of implicit elements such as Pavlovian and instrumental associations and associative conditioning; as well as explicit representations and cognitive predictions^4^.

These three elements of reward are linked but are dissociable both experimentally and in terms of their neural substrates. While this framework helps us understand how the organism responds to one reward at a time, how does the individual organism respond when confronted with more than one potential reward? According to a widely accepted model in neuroeconomics, the central stage in behavioral selection involves computing subjective values of all options on offer. This computation is thought to entail evaluating and weighting different attributes of each option, leading to a single comparable value, often denoted as goal value^5^, with the option with the highest goal value can then be selected.

Much of this work on the neural basis of reward has been derived from elegant studies in animal neuroscience. A challenge in investigating hedonics, goal values and motivation in humans has been the difficulty in measuring these different components reliably and objectively. It is critical to go beyond subjective measures of reward value such as self-ratings of liking or goal value, as might, for example, be recorded on a visual analogue rating. Given the difficulty of correctly introspecting about value and the doubts over whether it is reported truthfully, it is essential to develop of robust quantitative tools that can be validated.

While the objective hedonic reactions observed in rodents are also seen in human infants^6^, these are difficult to assess in adult humans. The liking or hedonic element thus remains a difficult one to measure objectively in adult humans. However it is possible to examine wanting or motivation more objectively and precisely and this paper describes a set of methods based on the use of grip force as a motivational measure. The amount of effort that individuals will expend to receive a reward is modulated by the magnitude of the reward they expect. The motivation towards the reward can be operationalized as the exerted effort. Pessiglione and colleagues elegantly demonstrated the use of grip force as a measure of motivation for monetary rewards, with participants exerting greater force for monetary rewards that had larger values^7^. In the case of food rewards the value of the food reward to the individual depends on several factors of which internal state (hunger or satiety) is a critical one^8^. With satiation, there is a decrease in neural activity in the orbitofrontal cortex (OFC), a brain region that encodes the current value of stimulus, to receipt of the same food^9,10^. The OFC projects to the ventral striatum which modulates motivational responding^7^. Thus a good motivational measure should show sensitivity to changes in reward value with satiation.

To determine goal value, direct subjective evaluations are nonideal. A more indirect way that also has the benefit of incentivizing participants to reveal their true goal value is to use a modified version of the Becker-DeGroot-Marschak auction^11^. Through a simple set of rules, this auction procedure motivates people to reveal the maximum amount of monetary resources they are willing to pay for items on offer. Their willingness to pay (WTP) is taken as measure of goal value.

## Protocol

All the procedures described in this protocol were developed and tested following ethical approval from the Cambridge Local Research Ethics Committees.

### 1. Grip Force as a Measure of Food Related Motivation

1. Apparatus and set up:
  1. Install MATLAB with Cogent toolboxes on the stimulus delivery laptop.
  2. Connect the force transducer and associated data acquisition system to the stimulus delivery laptop via the ethernet port. See **Table 1** for details of the required hardware and software. Note: To run the task in the magnetic resonance imaging (MRI) scanner a simple clench force rubber bulb is used, linked to pressure transducer outside the scanner. Changes in air pressure inside the bulb when it is squeezed, can be measured by the transducer and be directly related to the exerted effort.
  3. Set up the program to continuously poll the data acquisition system and read in 10 samples on each polling. Take the mean of the samples as the force exerted. Note: Galvanic skin response (GSR) can also be measured alongside. For this the stimulus delivery laptop requires a parallel port to connect to the GSR data acquisition system to synchronize the GSR with the other measures. Use a separate computer to record the measured GSR signal to minimize the load on the stimulus delivery laptop. GSR measurement is not discussed further.

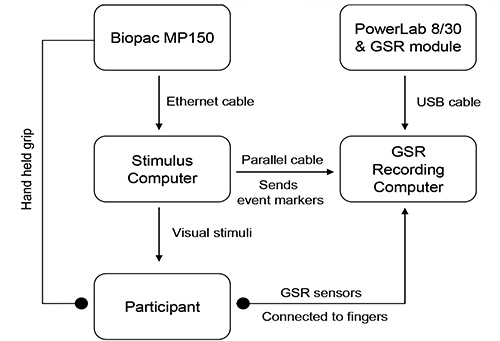
 **Figure 1. Hardware set up for the grip force task.**

1. Procedure:
  1. Set up the equipment on a table or other stable surface as shown in **Figure 1**. Wait for the data acquisition system to connect to the laptop.
  2. Collect a baseline measure for the grip force transducer and the participant's maximal effort, hereafter referred to as the maximal voluntary contraction (MVC) prior to starting the task.
    1. Place the grip force transducer on the table and collect the baseline measure.
    2. To measure the MVC, ask the participant to hold the grip force transducer and choose a comfortable arm position to maintain for the duration of the task. Instruct the participant to squeeze the transducer five times as hard as possible and take the mean of the five exertions as the maximal calibration for the task.Note: The force exerted by the participant during the response period is measured as a percentage of the difference between the baseline and the MVC as follows:
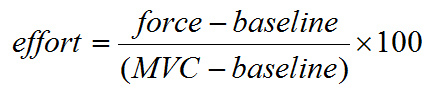
If the effort is negative it is set to the minimum force of zero and if it is greater than the MVC measured during calibration it is set to the maximum effort of 100 units. This is done to account for any remaining noise after the 10 sample point smoothing is applied as described above. The resulting series of effort samples are accumulated during the response period and stored as the effort response for the trial.
  3. Start the task. Note: If the task session is long, then fatigue effects can be taken in to account by measuring the MVC at the end of the task as well, and relating the fatigue effect to the difference between the MVCs at the beginning and the end of the task session.
2. Key dependent variables: This method captures the force-time curve on each trial. From the force-time curve extract the following variables: area under the curve (AUC), maximum or peak force exerted and the rate or the slope of the force time curve.
3. Applications: Three applications of the grip force measure are described below.
  1. Examining relative reward value: the Effortful Picture Selection task. Note: This task measures the relative effort participants are willing to exert to view different kinds of rewarding images such as foods and consumer goods. The effort ratings are related to their subjective liking ratings for the same images. The experimental design is as follows:
    1. Present two images side by side on each trial of the task; one is a large (300 x 300 pixels) and clearly visible default image, and the other a small (5 x 5 pixels) indistinct nondefault image. Exerting force on the transducer enlarges the nondefault image and shrinks the default image.
    2. Select 6 images from three reward categories: high calorie food, low calorie food and (gender specific) rewarding nonfoods. Have the images independently rated for subjective liking by a healthy volunteer group. Create image pairs such that every image is paired with all the images from the other two categories. Counterbalance the pairs such that each image appears as default and nondefault image in an equal number of trials.
    3. Running the task: Perform all steps described in section 1.2.
    4. Explain the trial structure to the participant and tell her that she can increase the size of the size of the nondefault image and correspondingly decrease that of the default image by squeezing the transducer. Note: The counterbalancing ensures that all images will have equal numbers of trials in which effort will enlarge or shrink them.
    5. Set the force required to make the nondefault image as large as possible at 10% of each individual's MVC (This percentage may have to be set individually for each Biopac module). Note: The relationship between exerted force and picture size is determined as follows. Assuming both the background and foreground images are square, let *lB* and *lF* be the length of background and foreground images respectively. Let *g, GMVC *and*, GBASE* be the grip force response measured during the response period of a trial, maximum force measured during calibration and baseline measured during calibration respectively. Further, assuming a linear relationship between grip force and image size, *lB* and *lF* can be written as
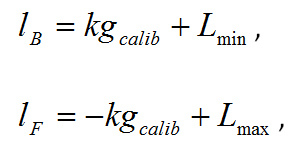
under the constraints
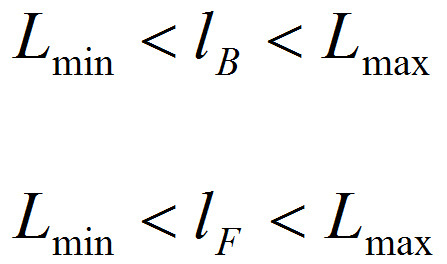
 where
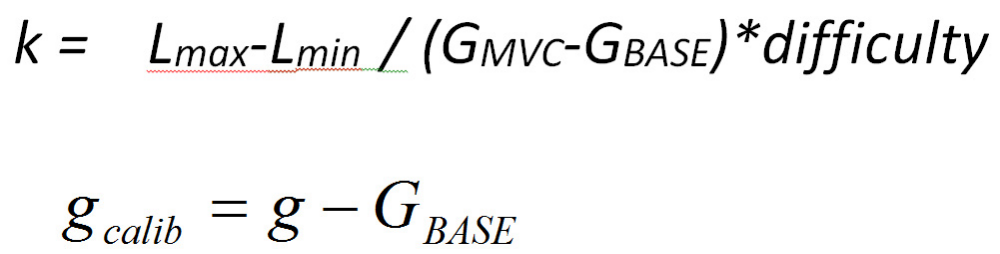
 The constraints ensure that an image does not expand more than Lmax even if a force exceeding GMVC is exerted.
    6. Present a short demonstration of the task.
    7. Tell the participant that she can view images as she choses depending on how much effort she exerts. Leave the participant on her own to complete the task.
    8. After the task is complete, collect liking ratings for each image on visual analogue scales subsequently.
    9. To analyze the data, for each image take the average of the areas under the force-time curves across all the trials in which it was the nondefault image and effort was required to enlarge it. This mean AUC is taken as the measure of the motivation for that particular image.
  2. Examining subliminal motivation for food. Note: Using both subliminal and conscious stimulus presentations, this task examines both subliminal and conscious motivation for food. The description below is based on a previous study using sensory specific satiation (see Ziauddeen *et al.*^16^). The experimental design is as follows:
    1. Select three stimuli: a savory food, a sweet food and a neutral nonfood item. Collect two images of each item, one for the conscious trial and the other for the subliminal trials so as to minimize direct motor specification effects^12^. Using Adobe Photoshop, format all images to have the same luminance and the same patterned background (a checkered array of 1 mm squares of yellow, red, green, and brown). Blur the picture edges using a single pass.
    2. Create a mask image by randomly scrambling all pictures, and then blend them to create a composite image.
    3. The subliminal presentation is achieved by sandwich masking. To do this present the mask image for 200 msec, then the stimulus image for 33 msec, followed by the mask again for 267 msec. For the conscious trials present the mask for 200 msec, then the stimulus image for 200 msec and the backward mask for 100 msec. The total presentation is 500 msec in both trial types.
    4. After the masked stimulus presentation, start the response window during which the participant can exert force on the grip force transducer. Provide real time feedback by way of a fluid level on the screen. Note: In all tasks using the fluid level for feedback to the participants, the height of the column and the maximal height that can be achieved are both varied randomly across trials. *e.g.* the height of the column is set to vary between 100, 110, and 120 units and the maximal height that can be achieved between 80, 90, and 100 units as a percentage of each participant's maximal force as measured at the start of the task. While the feedback serves an important role in engaging subjects with the task, this set up ensures that there is no consistent relationship between the exerted force and the height of the fluid level from trial to trial. The purpose is to prevent participants from modulating their effort to achieve a particular feedback, such as the getting the fluid level to the very top. Participants are explicitly informed that the feedback is unreliable and are instructed to judge their effort themselves and not to rely on the fluid level.
    5. For each trial, present a fixation cross for 500 msec, followed by the masked stimulus for 500 msec and then the response window with fluid level feedback for 3,000 msec.
    6. Running the task: Perform all steps as described in section 1.2.
    7. On each trial ask the participant to focus her attention on the central fixation cross. Explain to her that when the fluid level appears she can squeeze the force transducer to win points towards the item just presented. Explain that on some trials the image may be difficult to see and suggest she follow her instincts. Emphasize that the feedback in unreliable.
    8. Run the task in as many blocks as required.
    9. Following the completion of the task, check how well the masking procedure worked. This can be done in two ways: by collecting the participant's subjective report and by performing a forced choice discrimination. In the latter, present the masked stimulus as in the main task, followed by two options and ask the participant to indicate which one was just presented. Present each image as many times as it was presented in each block of the main task.
    10. Calculate the discriminability index (d') as per signal detection theory, if the masking has worked well, the d' should be close to zero.
  3. Examining food related motivation in the scanner.
    1. Using the grip force bulb and the accompanying pressure transducer, these grip force based tasks can be run in the MRI scanner. If feedback is used, then do so as in section 1.4.2.

### 2. Willingness to Pay as a Measure of Reward Value

Note: This task can be programmed in any appropriate stimulus delivery software and requires only a standard desktop or laptop computer. The version described here has been programmed in Presentation (version 14.5, Neurobehavioral Systems).

1. Procedures: Note: This procedure is a modification of the computerized auction procedure from Plassmann ^13^. The auction procedure is as follows:
  1. The auction involves a series of rounds, each featuring one food item. Photograph all food items on identical plates and backgrounds. Prior to the start of the task show participants the actual plates used in the images to provide an accurate sense of scale.
  2. Give the participants a fixed monetary budget, *e.g.* £3.
  3. Tell the participant that she will be taken through several rounds of the auction and she can place a bid on each round. She can place her bid on a sliding scale that goes from £0-£3 in increments of 10p.
  4. Inform the participant that one round will be selected at the end of the auction as the round that counts. Therefore she does not have to spread her £3 budget across different rounds, and can treat every round as if were the only one.
  5. Tell the participant the computer will be bidding against them in each round. If they outbid the computer on the selected round, they win the food item and only have to pay the amount the computer bid and will be able to keep any remaining change. If however, the computer outbids or matches their bid, they do not get the food item, but still get to keep their monetary budget.
  6. Explain to the participant that that given these set of rules, the best strategy for bidding in this auction is to bid the amount closest to how much they would be really willing to pay for the food item on offer. Note: The bid collected in this way corresponds to their WTP.
2. Applications:
  1. Examining sensitivity of the measure to changes in internal state. Note: As a proof of concept, the sensitivity of the measure to changes in value with changes in hunger and satiety was examined. Healthy normal-weight volunteers took part in the auction procedure before and after eating a fixed-calorie meal (550 kCal). A sensory specific satiety manipulation was used.
    1. Collect hunger and fullness ratings on a visual analogue scale.
    2. Perform the first block of the task as described in section 2.1. Design the task such that the planned food item is selected as the round that counts provided a nonzero bid is placed.
    3. Present the participant with the food they have won and give them 15 min to consume the meal. Subtract the payment from the £3 budget.
    4. Collect hunger and fullness rating again.
    5. Perform the second block of the task with a new £3 budget. No food is won in the second block.
    6. Contrast the WTP for different items across the two blocks.
  2. Future applications: Examining neural systems involved in value computation. Note: This task can be set up and run in the fMRI scanner to examine the neural correlates of value computation. It has recently been used in a pharmacological fMRI study looking at the effect of dopaminergic influences on value computation for food reward. Participants performed a single block of the task after receiving a single dose of either a dopamine agonist (bromocriptine), a dopamine antagonist (sulpiride) or placebo. The procedure was almost identical to section 2.1 apart from the fact that the scale was set to increase in 20p increments. Compared to similar tasks^13^, this measure permits the capture of the WTP as a more continuous variable as opposed to a discrete variable that takes a maximum of 4 values (corresponding to each button of the standard MRI button box).

## Representative Results

Representative results from the different applications of each of the methods described above are shown here:

### Grip force as a measure of motivation

*Examining relative reward value: the Effortful Picture Selection task*

This particular task was used in a proof-of-concept study of a novel mu-opioid antagonist drug GSK1521498 developed for the treatment of binge-eating and obesity^14^. In this study^15^, obese binge eating subjects were randomized to receive 4 weeks of either placebo or GSK1521498 at 2 mg/day or 5 mg/day. The aim was to determine whether the effects of the drug would be specific to food rewards without affecting other types of reward. **Figures 2** and **3** show the data from the placebo and the 5 mg/day groups for the food and nonfood rewards respectively. The key question in this study was whether the drug would have a specific effect on food rewards and this measure allowed us to examine this. **Figure 2** shows a specific effect of the drug on the motivation for high calorie food and it can be seen from **Figure 3**, there was no effect on the rewarding nonfood images.


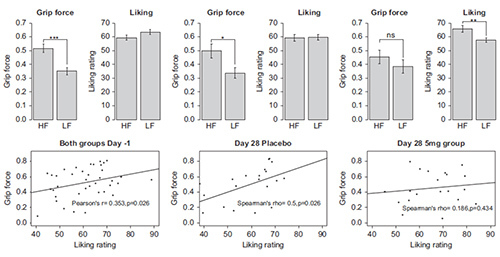
**Figure 2. Effortful Picture Selection task: effort expended and liking ratings for food images.** The top panel shows the grip force exerted and the corresponding liking ratings at baseline in both groups and separately for placebo and the GSK1521498 group at the end of treatment. It can be seen that the difference between the force exerted for high-fat (HF) versus low-fat (LF) food images is no longer significant after drug treatment, even though the subjective liking for these images is higher. The bottom panel displays the correlations between the exerted force and liking ratings for the high-fat food images, again at baseline for both groups and separately at Day 28.The correlation between these two measures is seen at baseline and in the placebo group at the end of treatment but is lost in the drug group (*p < 0.05; **p <  0.01; ***p < 0.001). This figure is reproduced from Cambridge *et al*.^14^). Please click here to view a larger version of this figure.


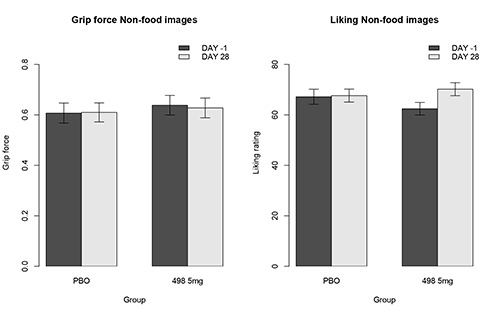
**Figure 3. Effortful Picture Selection task: effort expended and liking ratings for nonfood images. ** The left panel shows the force exerted to view rewarding nonfood images in the placebo (PBO) and GSK1521498 (498 5 mg) groups. The right panel shows the liking ratings for the same images. It can be seen that there are no significant drug effects on the motivation towards or subjective liking for rewarding nonfood images. This figure is reproduced from Cambridge *et al*.^14^).

*Examining subliminal motivation*

This particular application is from a previous study in which participants performed two blocks of a food incentive force task^16^. In between blocks they were specifically sated (sensory specific) on one of two test foods, all participants were given 30% of their daily calorie intake in this one eating episode**. **On each trial they had the opportunity to win points towards the prize at stake depending on the amount of force they exerted on a grip force transducer. There were conscious trials, in which the prize at stake was presented for 200 msec and subliminal trials, where it is only presented for 33 msec. There were two food prizes, pizza and cake, and one nonfood prize which served as a control item. The subliminal presentation was achieved using a sandwich masking procedure employing a forward and backward mask. For the analysis of the data, the dependent variable of interest was grip force, which was extracted as the area under the force curve (AUC) for all trials. To allow comparisons across all participants, for each subject, the AUC for each trial type was normalized by the maximum mean AUC of the 6 types (sated, nonsated, nonfood; both fed and fasted). This provided a normalized score between 0-1 for each participant. To control for fatigue, post-prandial sluggishness and nonspecific effects across the fed and fasted sessions, the AUC for the nonfood item in each session was used as a baseline and subtracted from the sated and nonsated measures. (For further details see Ziauddeen* et al.*^16^). It can be seen that the motivation of the sated food decreases in both conscious and subliminal conditions (see **Figure 4**).


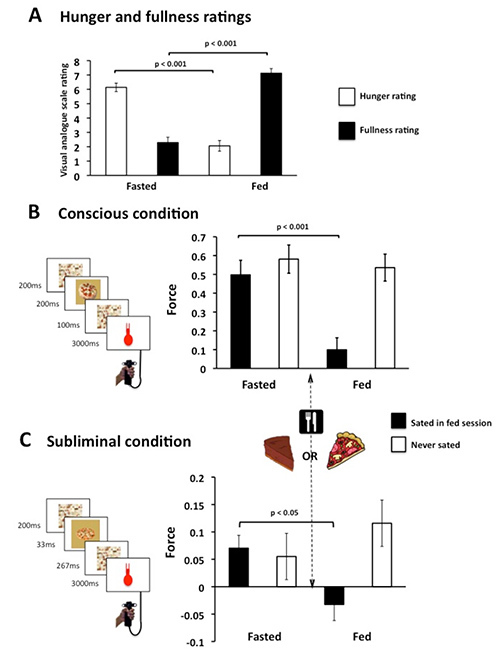
**Figure 4. Specific satiety modulates motivational effort. **(**A**) Change in hunger and fullness ratings with food consumption. (**B, C**) Participants exerted less for the food just consumed but still squeezed for the other food, regardless of awareness. Y-axis is the area under the curve normalized within subject and corrected for baseline changes. This figure is reproduced from Ziauddeen *et al.*^15^). Please click here to view a larger version of this figure.

*Examining food related motivation in the scanner*

Participants performed a session of the incentive force task in the scanner in which they squeezed the clench bulb to win points towards high calorie and low calorie food items for their lunch after the session. **Figure 5** presents illustrative data from 10 subjects in a recent pharmacological fMRI study that shows that the task captures differential motivation for highly rewarding versus less rewarding foods.


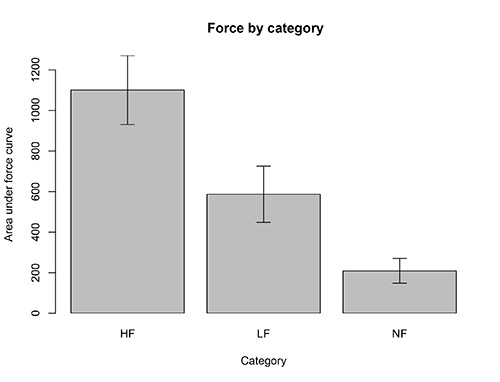
**Figure 5. Force exerted for different reward categories.** Subjects squeezed the grip force bulb in the scanner to win points towards items from 3 different categories (HF: high calorie foods, LF: low calorie foods, NF: neutral nonfoods).

**Willingness to pay as measure of reward value**

**Figure 6** presents the findings from the proof of concept study of the effect of satiety on willingness to pay for food rewards. To facilitate the within subjects comparison, each subject's bids were normalized by dividing by their maximum bid. It can be seen that participants were willing to pay more for the food items in the first round. This was reduced by satiation with 600 calories of the test food as reflected in lower bids in the second round. There appeared to be a stronger effect of satiation with savory foods compared to sweet foods but this was not significant.


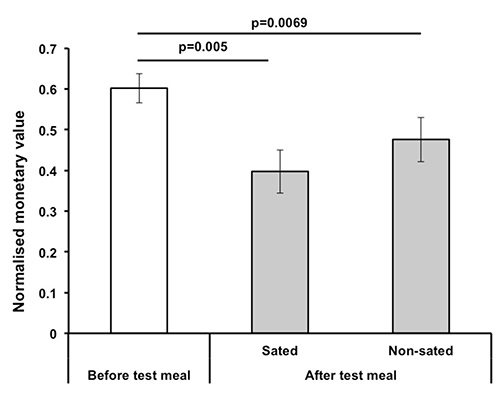
**Figure 6. Effect of satiety on willingness to pay (WTP).** 10 healthy volunteers took part in this study. It can be seen that the normalized mean bid decreased following satiation with the study meal. As a sensory specific satiety manipulation was used the results for the second auction are presented separately for foods from the sated and nonsated categories. While there appears to be a greater effect on foods from the sated category, this was not statistically significant.

## Discussion

This article describes a set of measures for the measurement of motivation for food rewards and reward value. The application of grip force measures to the study of food related motivation in humans is particularly novel. Representative results have been presented for most of the applications described demonstrating the value of these methods and their sensitivity to metabolic and pharmacological manipulations and therefore their potential further use in future studies of food related motivation and in trials of anti-obesity drugs. These more objective measures are easily transferable and comparable across different settings. It would also be useful to consider the influence of individual variability on these measures by examining the effects of traits such as impulsivity on them.

Some important points must be emphasized. In the use of the grip force tasks, the initial calibration and the capture of the MVC are critical. It is ideal to capture the grip response from the start of each trial and not limit it solely to the designated response window as this permits the examination of premature responding. There is some variability in the Biopac MP150 modules so each module should be tested to determine the settings that are most suitable for that piece of equipment. Where nonstandardized images are used, it is critical to have them independently rated to determine how rewarding they are perceived to be. An important limitation of all these measures is that they do not capture liking for the food rewards used so it is important to collect measures of subjective liking using visual analogue scales or other appropriate tools, after the task. These can then be compared to the motivational measures or used to model the effects of other variables on the primary dependent variables. However it is important to note that in studies where actual food rewards are consumed, liking ratings collected after the task are likely to be affected by food consumption. This emphasizes the importance of having not only representative images but also representative foods *e.g.* a chocolate brownie that looks and tastes like most chocolate brownies would; and to have these independently rated.

Finally it is critical to acknowledge that all these measures use food pictures as representations of food rewards instead of actual food rewards. While this does present some limitations, anticipatory behavior towards food and decisions regarding food are often guided by representations of the food rather than by immediate consummatory reward. Nevertheless, it is essential for the methods described here that when they are linked to outcomes such as successfully bidding for a food, real outcomes should be delivered.

## Disclosures

The authors declare that they have no competing financial interests. HZ, NS, VCC, ISF, and PCF have been involved in academic collaborations with GlaxoSmithKline that have used some of the techniques described in this manuscript.

## References

[B0] Swinburn BA (2011). The global obesity pandemic: shaped by global drivers and local environments. Lancet.

[B1] Berthoud H-R, Lenard NR, Shin AC (2011). Food reward, hyperphagia, and obesity. AJP Integr. Comp. Physiol.

[B2] Rolls ET (2007). Understanding the mechanisms of food intake and obesity. Obesity Rev.

[B3] Berridge KC, Robinson TE, Aldridge JW (2009). Dissecting components of reward: 'liking', "wanting," and learning. Curr. Opin. Pharmacol.

[B4] Rangel A, Camerer C, Montague PR (2008). A framework for studying the neurobiology of value-based decision making. Nat. Rev. Neurosci.

[B5] Steiner JE, Glaser D, Hawilo ME, Berridge KC (2001). Comparative expression of hedonic impact: affective reactions to taste by human infants and other primates. Neurosci. Biobehav. Rev.

[B6] Pessiglione M (2007). How the Brain Translates Money into Force: A Neuroimaging Study of Subliminal Motivation. Science.

[B7] Berridge KC (2004). Motivation concepts in behavioral neuroscience. Physiol. Behav.

[B8] Kringelbach ML, O'Doherty J, Rolls ET, Andrews C (2003). Activation of the human orbitofrontal cortex to a liquid food stimulus is correlated with its subjective pleasantness. Cerebral Cortex.

[B9] Valentin VV, Dickinson A, O'doherty JP (2007). Determining the Neural Substrates of Goal-Directed Learning in the Human Brain. J. Neurosci.

[B10] Becker GM, DeGroot MH, Marschak J (1964). Measuring utility by a single-response sequential method. Behav. Sci.

[B11] Neumann O (1990). Direct parameter specification and the concept of perception. Psychol. Res.

[B12] Plassmann H, O'Doherty J, Rangel A (2007). Orbitofrontal cortex encodes willingness to pay in everyday economic transactions. J. Neurosci.

[B13] Cambridge VC (2012). Neural and Behavioral Effects of a Novel Mu Opioid Receptor Antagonist in Binge-Eating Obese People. Biol. Psych.

[B14] Ziauddeen H (2012). Effects of the mu-opioid receptor antagonist GSK1521498 on hedonic and consummatory eating behaviour: a proof of mechanism study in binge-eating obese subjects. Mol. Psych.

[B15] Ziauddeen H, Subramaniam N, Gaillard R, Burke LK, Farooqi IS, Fletcher PC (2011). Food images engage subliminal motivation to seek food. Int. J. Obesity.

